# An Atypical Clinical Presentation of Post-traumatic Syringomyelia: A Case Report and Brief Review of the Literature

**DOI:** 10.7759/cureus.1852

**Published:** 2017-11-16

**Authors:** Muhammad Uzair Lodhi, Aaron R Kuzel, Intekhab Askari Syed, Mustafa Rahim

**Affiliations:** 1 Medical Student, Department of Medicine, Raleigh General Hospital, Beckley, Wv; 2 Department of Emergency Medicine, Lincoln Memorial University-Debusk College of Osteopathic Medicine; 3 Medical Student, Department of Medicine, Beckley Appalachian Regional Healthcare; 4 Assistant Clinical Professor of Internal Medicine, West Virginia University School of Medicine

**Keywords:** loss of heat and pain sensation, atypical-syringomyelia, non-dissociated sensory loss, loss of fine-touch, syrinx formation from c2-t1, dysesthetic pain

## Abstract

Syringomyelia classically presents as a bilateral sensory loss of the dissociated type which includes the loss of pain and temperature with the preservation of fine touch, vibratory sensation, and proprioception in the shoulder, arm, and hand. Eventually, weakness of the legs, muscle wasting, and ataxia can also be seen due to the involvement of the corticospinal tracts and the posterior columns of the spinal cord. We present the case of a 64-year-old patient with an atypical presentation of post-traumatic syringomyelia. This atypical presentation included a unilateral sensory loss of fine touch, pain, and temperature in the shoulder, arm, and hand which was of the non-dissociated type with no weakness, muscular atrophy, loss of vibratory sensation, or proprioception.

## Introduction

Syringomyelia is a cavitary expansion or formation of a syrinx in the central canal of the spinal cord. The majority of cases are due to the obstruction of cerebrospinal fluid (CSF) circulation caused by an Arnold-Chiari malformation or tumors. The remaining small percentage of reported syringomyelia cases is either idiopathic or develop following an injury to the spinal cord. Syringomyelia classically presents as a loss of pain and temperature sensation in the neck, shoulder, and bilateral upper extremities with the preservation of fine touch, vibratory sensation, and proprioception [1]. As the syrinx expands and compresses the spinal nerves, pyramidal signs appear in the lower extremities together with weakness and muscular atrophy in the neck, upper back, and all other extremities [1]. The majority of the cases with syringomyelia are also associated with severe dysesthetic pain in the neck, shoulders, and the back. The symptoms can present anytime from six months to 34 years after the initial trauma [2]. The syrinx can expand cranially, caudally, or in both directions [3]. Some studies have shown that risk factors such as increased age (at the time of spinal cord injury), severity, and cervical location of the injury have a significantly higher chance of developing a syrinx within five years of the injury [3].

In this report, we describe a patient with paresthesias in the neck and shoulders, along with the loss of fine touch, pain, and temperature in the left upper extremity. There was no muscular atrophy, pyramidal signs, loss of vibratory sensation, or proprioception in any of the extremities which makes this case a very atypical presentation of syringomyelia.

## Case presentation

A 64-year-old male, a former coal miner, presented to the clinic with severe pain and paresthesias in his neck and shoulders. On careful examination, it was found that the patient had been dealing with this pain since the age of 49 following an uncomplicated traumatic head injury in the coal mine which resulted in a crushed helmet. He had been experiencing pain and paresthesias which were progressively getting worse especially over the period of the last few years. The pain was described as a 'burning-stretchy' sensation aggravated by a sudden change in posture such as standing or sitting. The patient tried narcotics, antiepileptics, and tricyclics but they offered no alleviation of his symptoms. The pain and paresthesias were followed by an associated loss of heat or cold sensation in the left upper extremity. Interestingly, the toes of the patient also become cyanotic when exposed to cold temperature. His past medical history was significant for systemic lupus erythematosus, peripheral vascular disease, hypertension, polyarthritis, and meningitis. No muscular weakness, loss of urinary or bowel control were reported. There was no significant family history reported. The physical examination exhibited blisters on his right upper extremity but the patient was not in acute distress. His vitals were as follows: afebrile, blood pressure of 122/72, heart rate of 57 beats per minute, and a respiratory rate of 17 breaths per minute. Upon neurological and musculoskeletal examination of the patient, the cranial nerves II-XII were found to be grossly intact and the higher cerebral function was normal. Loss of fine touch, pain, and temperature sensation was noticeable in the C3-T1 dermatomes. However, sensations of crude touch and pressure were intact. The Romberg sign was negative but there was some notable heel/shin ataxia. Reflexes were 2+ bilaterally for biceps, triceps, brachioradialis, patellar, and the achilles. In addition to the absence of any of the pyramidal signs, no weakness was noted in both the upper or lower limbs. Radiofemoral delay was also not present. Additionally, the physical examination of the other systems was unremarkable.

**Figure 1:**
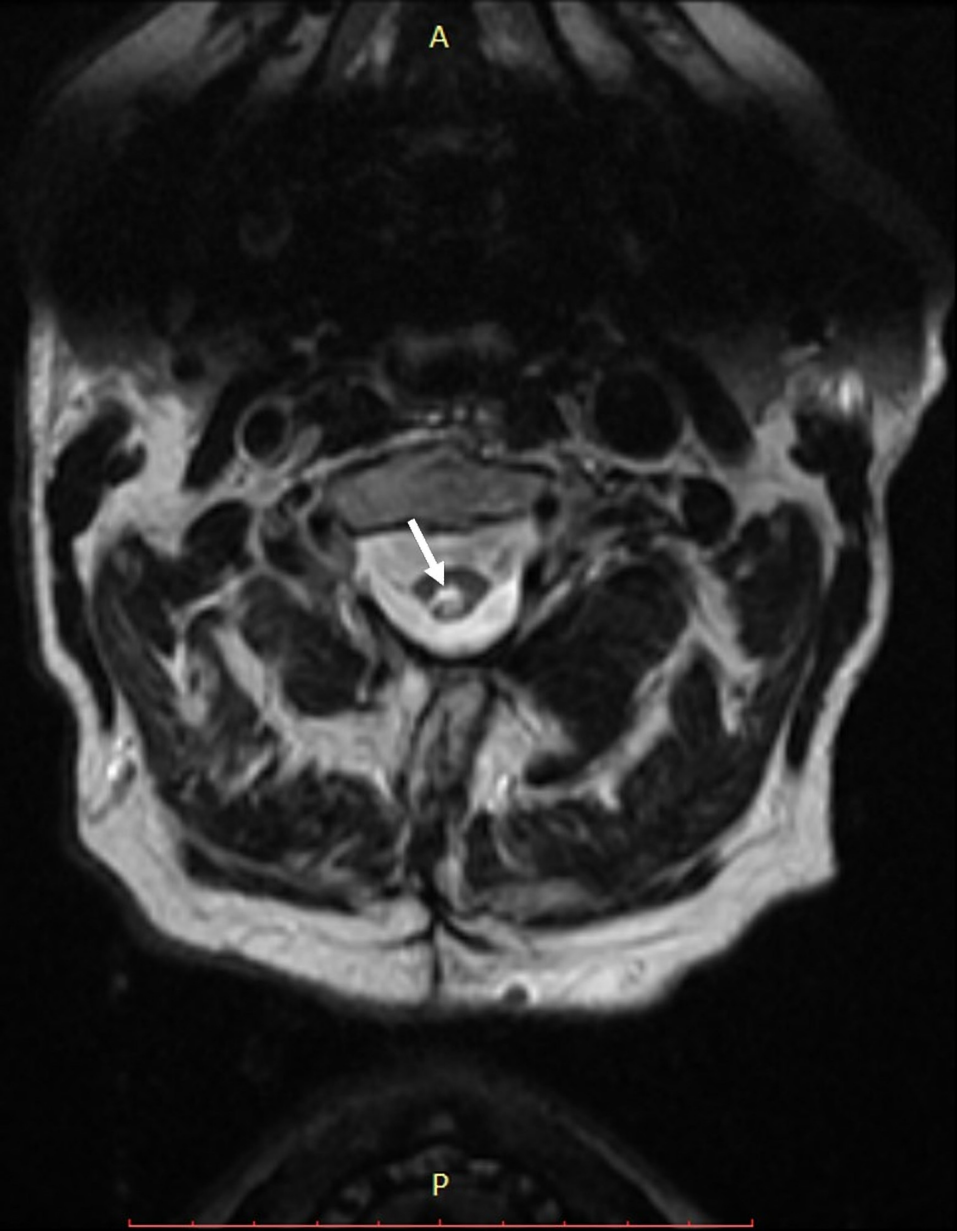
Axial T2-weighted magnetic resonance imaging (MRI) at the level of C2 Showing hyperintense fluid in the spinal cord (white arrow) consistent with a syrinx.

**Figure 2:**
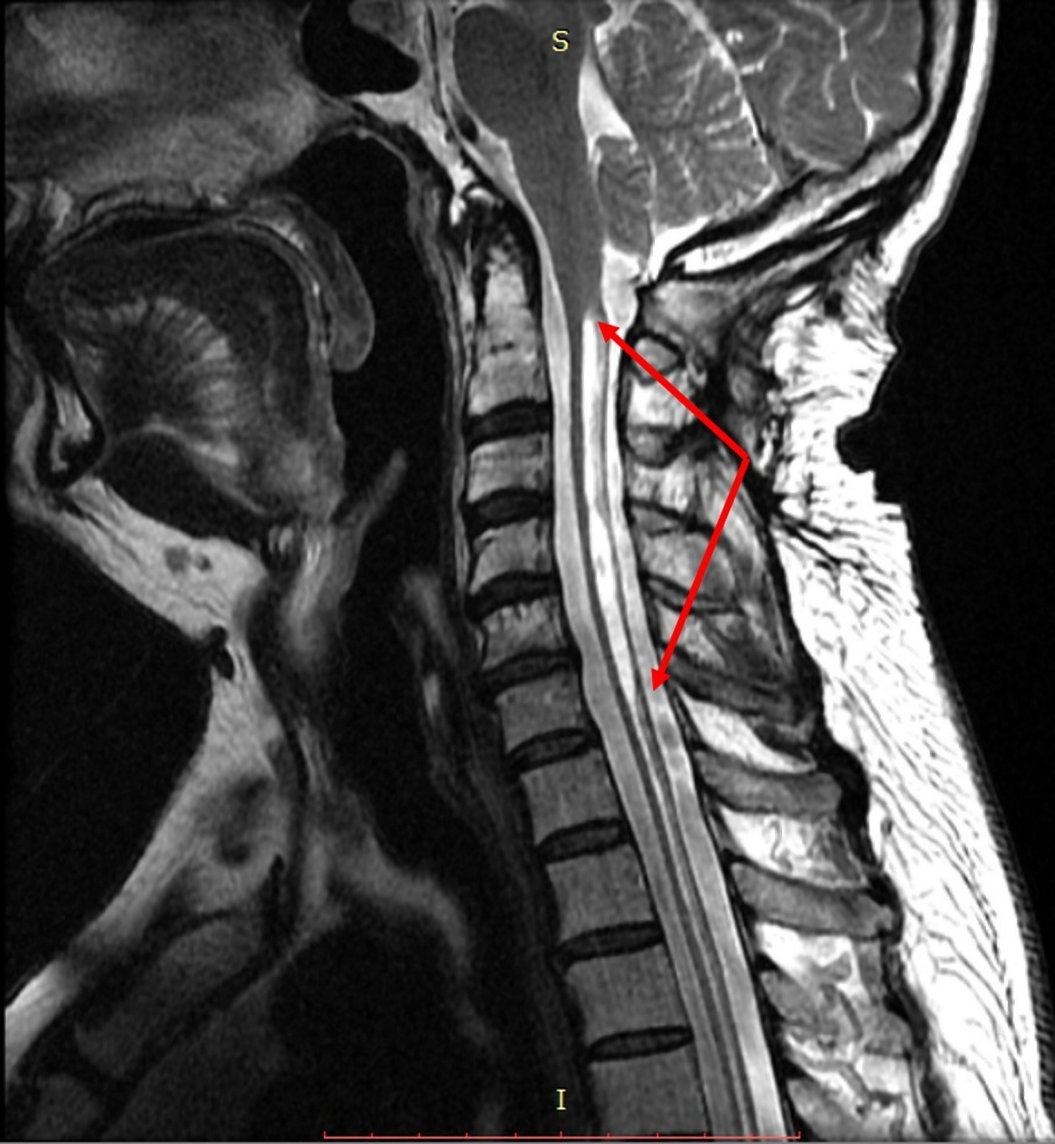
Sagittal T2-weighted magnetic resonance imaging (MRI) of the spine Showing syrinx formation (hyperintense region) from C2-T1, with the greatest dilation around C2-C7 (red arrows).

Routine biochemical and hematologic studies, including vitamin B12, thyroid, and serologic testing for syphilis was unremarkable. The radiological survey of the patient including a computerized tomography (CT) scan showed no abnormalities. However, the magnetic resonance imaging (MRI) scan of the cervical and thoracic spine showed dilation of the cavitary longitudinal dilation from C2-T1 with the greatest dilation from C2-C7 (Figures 1-2).

## Discussion

The exact pathophysiology of post-traumatic syringomyelia is not known. However, some factors that are thought to precede the initial formation of the cavity within the spinal cord include mechanical compression of the spinal cord, inflammation of the spinal cord, hematoma, release of intracellular lysosomal enzymes, ischemia, and obstruction of the arterial and venous supply [4]. All these factors are thought to contribute towards syrinx formation. The syrinx eventually expands and causes the destruction of the spinothalamic tracts, causing a classic cape-like bilateral distribution of pain and temperature loss in the shoulders, arms, and hands. The syrinx often doesn’t affect the dorso-lateral column of the spinal cord, leaving the sensation of fine touch, vibration, and proprioception intact [5].

The classical dissociated type of sensory loss in syringomyelia is due to the location of the syrinx in the central canal of the spinal cord, resulting in disruption of decussating spinothalamic tracts [5]. However, the location of the syrinx within the spinal cord can be variable; if it is leaning more towards the dorso-lateral, then the minor distructions to the posterior column of the spinal cord can produce diverse and an atypical sensory loss [5]. Our patient with post-traumatic syringomyelia presented with an atypical clinical presentation of loss of pain, temperature, and fine touch in the left upper limb, with no weakness or pyramidal signs, in addition to severe dysesthetic pain in the neck, shoulder, and the upper back. The unusual non-dissociated type of sensory loss could be explained by the expansion of the syrinx affecting the posterior columns of the spinal cord in addition to the lateral spinothalamic tracts. Another explanation could be the syrinx predominantly compressing the dorso-lateral region of the spinal cord [5]. Additionally, the absence of pyramidal signs can be explained by sparing of the corticospinal tracts. 

The patient went through a surgical technique of laminectomy with direct drainage of the syrinx which alleviated the dysesthetic pain for a few months. However, all the symptoms were back again with the recurrence of the syrinx in a very short period of time. The next possible step could be syringo-peritoneal, syringo-pleural, or a syringo-subarachnoid shunt; however, it is controversial whether the placement of shunts would be a superior alternative for patients with post-traumatic syringomyelia [6-7]. The result of surgeries for post-traumatic syringomyelia is not nearly as pleasing as they are for a patient with hindbrain-related syringomyelia. However, even modest improvement or halting the further neurological deterioration of a patient for a short period of time with surgery can dramatically enhance the quality of life and also reduce the burden on health care costs [8].

Lastly, our patient also had multiple episodes of meningitis in the following years after the spinal trauma. Meningitis is reported to cause spinal adhesive arachnoiditis and can lead to the formation of a syrinx due to the disruption of CSF flow [9]. However, in our case, meningitis was ruled out as the cause of syringomyelia due to the absence of any significant adhesive arachnoiditis in the MRI.

## Conclusions

In this case report, we tried to focus on the variability in the presentation of syringomyelia. If a clinical diagnosis is solely based on the presence of a dissociated type of sensory loss, then the chances of missing syringomyelia in many cases are high. Even patients presenting with a non-dissociated type of sensory loss, in the absence of any pyramidal signs, should be evaluated carefully for the possibility of syringomyelia. In addition, further research is needed to enhance the treatment options of post-traumatic syringomyelia in order to improve the quality of life for patients with the primary focus on the reduction of syrinx reformation after surgery.
